# Supplementary material for: The Conservation of Long Intergenic Non-Coding RNAs and Their Response to Verticillium dahliae Infection in Cotton
Source: Int J Mol Sci. 2022 Aug 2;23(15):8594. doi: 10.3390/ijms23158594 (PMC9368808; doi:10.3390/ijms23158594)
Supplement: Supplementary file 1 [file ijms-23-08594-s001.zip › Supplemental Fig_S1_to_S5.pdf]

Supplemental Figure S1. The overall flowchart showing the analyses and methodology applied in the study.

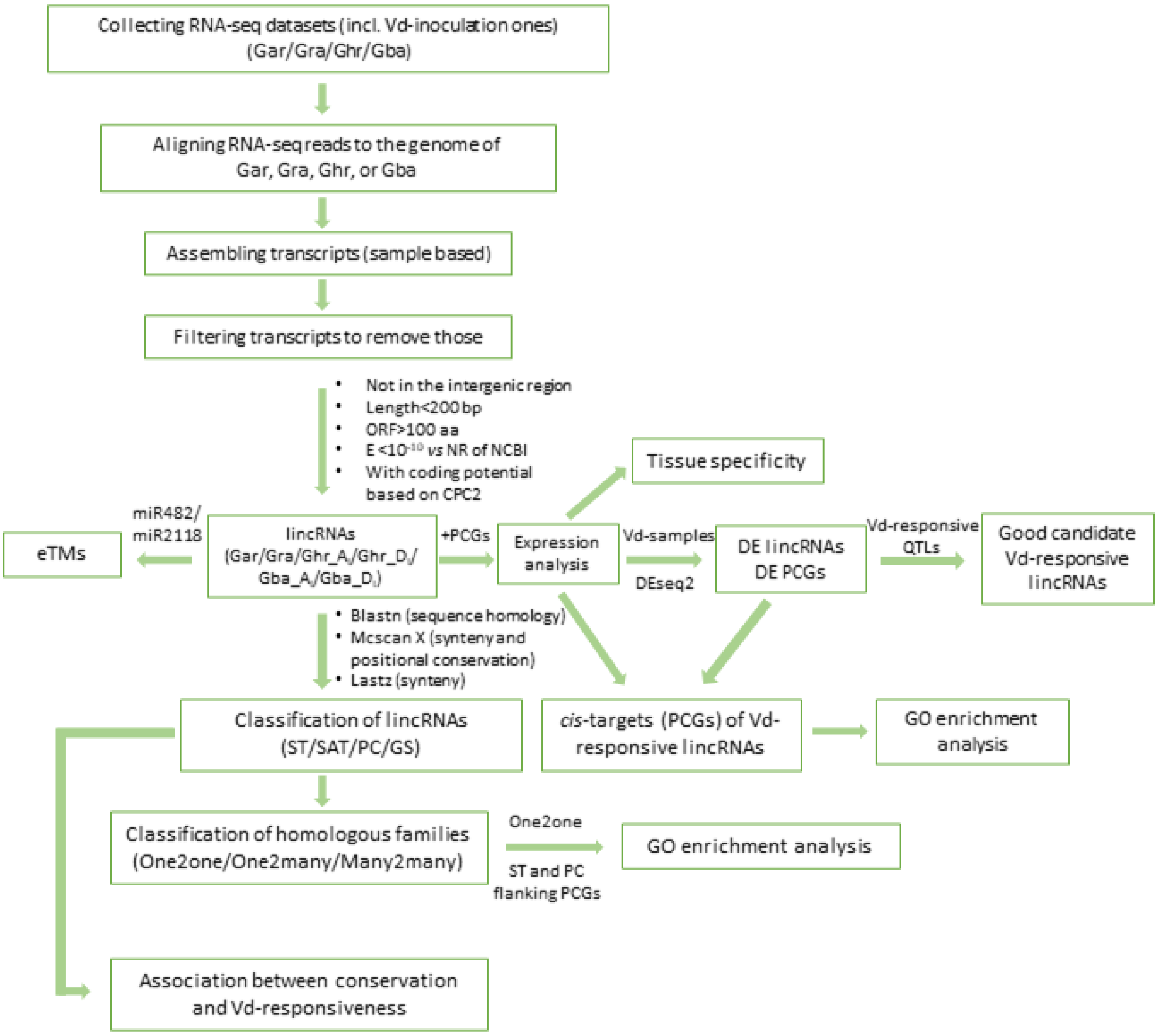

Supplemental Figure S2. Distribution of neighbouring protein coding genes (A) and lincRNAs (B) in the genome of *G. hirsutum*.

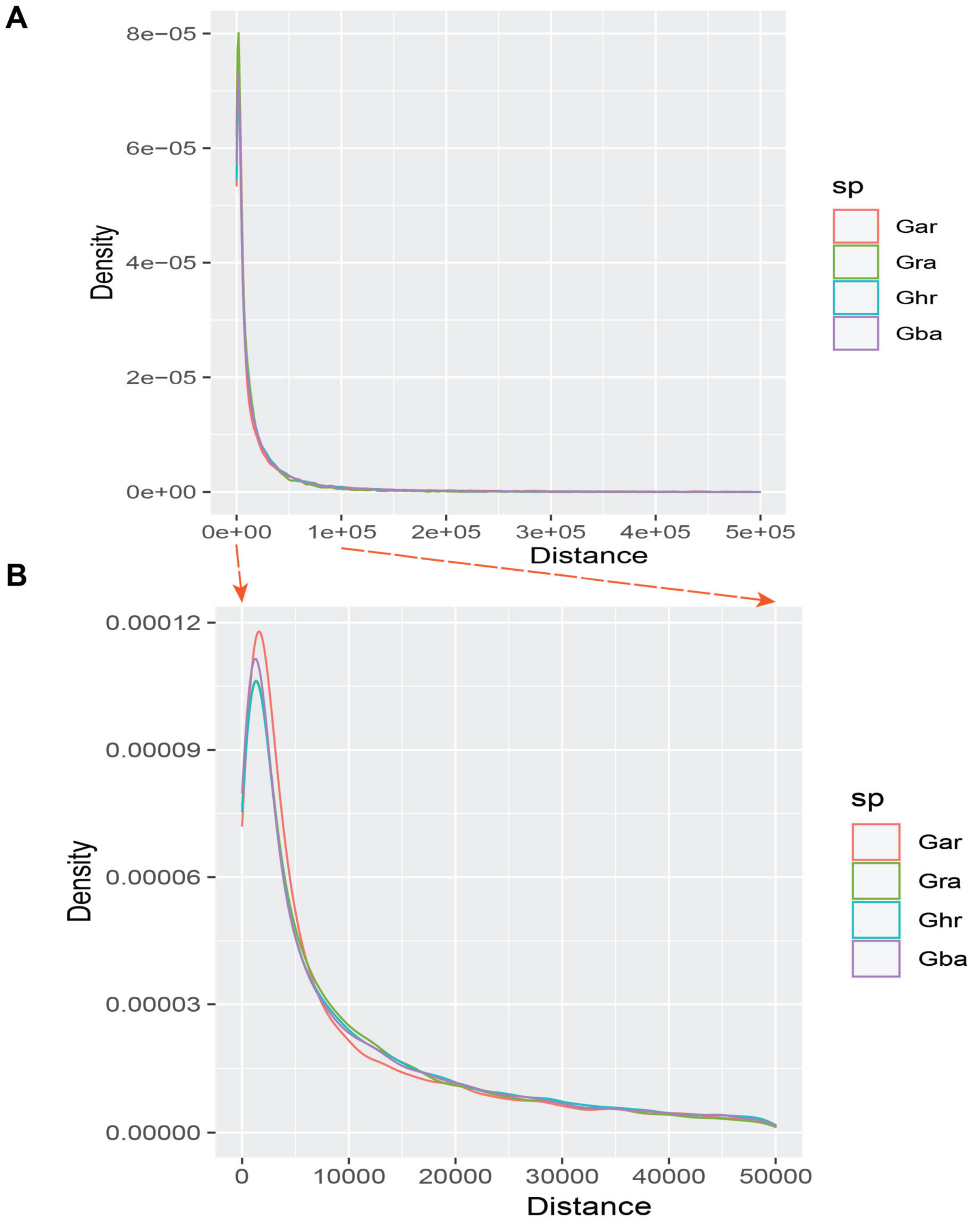

Supplemental Figure S3. Sequence alignment of Ghrlnc.30744 with its homologs from other five (sub)genomes and the two full-length cDNAs generated by PacBio SMART sequencing. While the length of the predicted lincRNAs is variable, the splicing event of the intron is conserved. The 2596-bp 3' end sequence of Garlnc.26799 is not shown as it is unique to *G. arboreum*.

```
PB.6980.1_gDNA      ------------------------------------------------------------     0
PB.6980.1           ------------------------------------------------------------     0
PB.23765.1          ------------------------------------------------------------     0
Ghrlnc.30744_At     CACTAGTGAAAACATTGTAAAAAATCTCCGCCGTC-CAAACAATTTCAATCACCCCCAAT     59
Garlnc.26799        ------------------------------------------------------------     0
Gbalnc.26265_At     ------------------------------------------------------------     0
Gbalnc.64928_Dt     ------------------------------------------------------------     0
Ghrlnc.74560_Dt     ------------------------------------------------------------     0
Gralnc.30550        ------------------------------------------------------------     0

PB.6980.1_gDNA      ------------------------------------------------------------     0
PB.6980.1           ------------------------------------------------------------     0
PB.23765.1          ------------------------------------------------------------     0
Ghrlnc.30744_At     CACG---------TCTCCTTTTCGTGTTGTAAAAAAGTGGAGAAAGTCATTATTATAGGA     110
Garlnc.26799        ------------------------------------------------------------     0
Gbalnc.26265_At     ------------------------------------------------------------     0
Gbalnc.64928_Dt     -------------------------------------GGAGAAAGTCATTATTATAGGAA     23
Ghrlnc.74560_Dt     ------------------------------------------------------------     0
Gralnc.30550        ------------------------------------------------------------     0

PB.6980.1_gDNA      ------------------------------------------------------------     0
PB.6980.1           ------------------------------------------------------------     0
PB.23765.1          ------------------------------------------------------------     0
Ghrlnc.30744_At     ATTGGATATGATCATACTCTGACACCCAAAAAACACATTTTTTTAATTATT---------     161
Garlnc.26799        ------------------------------------------------------------     0
Gbalnc.26265_At     ------------------------------------------------------------     0
Gbalnc.64928_Dt     TTGGATATGATCATACTCTGACACCCAAAAAACACTTTTTTTTTAATTATTATTAATAAT     83
Ghrlnc.74560_Dt     ---------------------------AAAAACACTTTTTTTTTAATTATTATTAATAAT     33
Gralnc.30550        -----------------------------------CTTTTTTTTAATTATTATTAATAAT     25

PB.6980.1_gDNA      ------------------------------------------------------------     0
PB.6980.1           ------------------------------------------------------------     0
PB.23765.1          ------------------------------------------------------------     0
Ghrlnc.30744_At     ATTAATAATTTAATAACACTCCTATTATTAGTAATTTTTAATTATATAATCATCATCGAA     221
Garlnc.26799        --------------------------------AATTTTTAATTATATAATCATCATCGAA     28
Gbalnc.26265_At     --------------AACACTCCTATTATTAGTAATTTTTAATTATATAATCATCATCGAA     46
Gbalnc.64928_Dt     ------AATTTAATAACACTCCTATTATTAGTAATTTTTAATTATATAATCATCATCGAA     137
Ghrlnc.74560_Dt     ------AATTTAATAACACTCCTATTATTAGTAATTTTTAATTATATAATCATCATCGAA     87
Gralnc.30550        ------AATTTAATAACACTCCTATTATTAGTAATTTTTAATTATATAATCATCATCGAA     79

PB.6980.1_gDNA      ------------------------------------------ACAGTGTTTCGGGAT--T     16
PB.6980.1           ------------------------------------------ACAGTGTTTCGGGAT--T     16
PB.23765.1          ------------------------------------------ACTGTATTTCGGGAT--T     16
Ghrlnc.30744_At     ACAAAGTCCAATTTATAAGCCATTTT-AGCCTCTTCTCCTTCACAGTGTTTCGGGAT---     277
Garlnc.26799        ACAAAGTCCAATTTATAAGCCATTTT-AGCCTCTTCTCCTTCACAGTGTTTCGGGAT---     84
Gbalnc.26265_At     ACAAAGTCCAATTTATAAGCCATTTT-AGCCTCTTCTCCTTCACAGTGTTTCGGGAT---     102
Gbalnc.64928_Dt     ACAAAGTCCAATTTATAAGC-ATTT-CAGCCTCTTCTCCTTCACTGTATTTCGGGAT--T     193
Ghrlnc.74560_Dt     ACAAAGTCCAATTTATAAGC-ATTT-CAGCCTCTTCTCCTTCACTGTATTTCGGGAT--T     143
Gralnc.30550        -CAAAGTCCAATTTATAAGC-ATTT-CAGCCTCTTCTCCTTCACTGTGCTTCGGGAT--T     134
                                                              ** **  ********

PB.6980.1_gDNA      --TCTCGTGCTCTGTTTTTTTATTCTTTGTTGAGAAAGAAG---GGATCCTTGCTTTGCT     71
PB.6980.1           --TCTCGTGCTCTGTTTTTTTATTCTTTGTTGAGAAAGAAG---GGATCCTTGCTTTGCT     71
PB.23765.1          --TCTCGTGCTCTGTTTTTTTATTCTTTGTTGAGAAAGAAG---GGATCCTTGCTTTGCT     71
Ghrlnc.30744_At     -TTCTCGTGCTCTGTTTTTTTATTCTTTGTTGAGAAAGAAG---GGATCCTTGCTTTGCT     333
Garlnc.26799        -TTCTCGTGCTCTGTTTTTTTATTCTTTGTTGAGAAAGAAG---GGATCCTTGCTTTGCT     140
Gbalnc.26265_At     -TTCTCGTGCTCTGTTTTTTTATTCTTTGTTGAGAAAGAAG---GGATCCTTGCTTTGCT     158
Gbalnc.64928_Dt     --TCTCGTGCTCTGTTTTTTTATTCTTTGTTGAGAAAGAAG---GGATCCTTGCTTTGCT     248
Ghrlnc.74560_Dt     --TCTCGTGCTCTGTTTTTTTATTCTTTGTTGAGAAAGAAG---GGATCCTTGCTTTGCT     198
Gralnc.30550        --TCTCGTGCTCTGTTTTTTTATTCTTTGTTGAGAAAGAAG---GGATCCTTGCTTTGCT     189
                      *****************    ******************   ****************

PB.6980.1_gDNA      TTGATTATTTTT-------CAATGATGATGGAGCTTTTTGTATTGGGGTGTACGGGAGTT     124
PB.6980.1           TTGATTATTTTT-------CAATGATGATGGAGCTTTTTGTATTGGGGTGTACGGGAGTT     124
PB.23765.1          TTGATTATTTTTT------CAATGATGATGGAGCTTTTTGTATTGGGGTGTACGGGAGTT     125
```

```
Ghrlnc.30744_At      TTGATTATTTTT-------CAATGATGATGGAGCTTTTTGTATTGGGGTGTACGGGAGTT    386
Garlnc.26799         TTGATTATTTTT-------CAATGATGATGGAGCTTTTTGTATTGGGGTGTACGGGAGTT    193
Gbalnc.26265_At      TTGATTATTTTT-------CAATGATGATGGAGCTTTTTGTATTGGGGTGTACGGGAGTT    211
Gbalnc.64928_Dt      TTGATTATTTTT-------CAATGATGATGGAGCTTTTTGTATTGGGGTGTACGGGAGTT    301
Ghrlnc.74560_Dt      TTGATTATTTTT-------CAATGATGATGGAGCTTTTTGTATTGGGGTGTACGGGAGTT    251
Gralnc.30550         TTGATTATTTTT-------CAATGATGATGGAGCTTTTTGTATTGGGGTGTACGGGAGTT    242
                     ************       *****************************************

PB.6980.1_gDNA       GTGG-TGTTCCTCCACGGTGCCAATTTCT-TCTTCCATGTTCTTT-CTCAACATC--TCG    181
PB.6980.1            GTGTGTGTTCCTCCACGGTGCCAATTTCTCTCTTCCATGTTCTTTTCTCAACATC--TCG    184
PB.23765.1           GTGG-TGTTCCTCCACGGTGCCAATTTCT-TCTTCCATGTTCTTT-CTCAACATCTCTCG    182
Ghrlnc.30744_At      GTGG-TGTTCCTCCACGGTGCCAATTTCT-TCTTCCATGTTCTTT-CTCAACATC--TCG    441
Garlnc.26799         GTGG-TGTTCCTCCACGGTGCCAATTTCT-TCTTCCATGTTCTTT-CTCAACATC--TCG    248
Gbalnc.26265_At      GTGG-TGTTCCTCCACGGTGCCAATTTCT-TCTTCCATGTTCTTT-CTCAACATC--TCG    266
Gbalnc.64928_Dt      GTGG-TGTTCCTCCACGGTGCCAATTTCT-TCTTCCATGTTCTTT-CTCAACATC--TCG    356
Ghrlnc.74560_Dt      GTGG-TGTTCCTCCACGGTGCCAATTTCT-TCTTCCATGTTCTTT-CTCAACATC--TCG    306
Gralnc.30550         GTGG-TGTTCCTCCACGGTGCCAATTTCT-TCTTCCATGTTCTTT-CTCAACATC--TCG    297
                     ***  ************************ *************** *********  ***

PB.6980.1_gDNA       CTGTTCGATCTCTC--AGGTTCGTTCCATTTTTT-------TCAACAATTTCTAT-----    227
PB.6980.1            CTGTTCGATCTCTCTCAG------------------------------------------    202
PB.23765.1           CTGTTCGATCTCTC--AG------------------------------------------    198
Ghrlnc.30744_At      CTGTTCGATCTCTC--AG------------------------------------------    457
Garlnc.26799         CTGTTCGATCTCTC--AG------------------------------------------    264
Gbalnc.26265_At      CTGTTCGATCTCTC--AG------------------------------------------    282
Gbalnc.64928_Dt      CTGTTCGATCTCTC--AG------------------------------------------    372
Ghrlnc.74560_Dt      CTGTTCGATCTCTC--AG------------------------------------------    322
Gralnc.30550         CTGTTCGATCTCTC--AG------------------------------------------    313
                     **************  **

PB.6980.1_gDNA       --------AATTTTCTCACAATTA-TACCATGTCCCAAATTGCAATAAATCTGGGTAAGC    278
PB.6980.1            ------------------------------------------------------------    202
PB.23765.1           ------------------------------------------------------------    198
Ghrlnc.30744_At      ------------------------------------------------------------    457
Garlnc.26799         ------------------------------------------------------------    264
Gbalnc.26265_At      ------------------------------------------------------------    282
Gbalnc.64928_Dt      ------------------------------------------------------------    372
Ghrlnc.74560_Dt      ------------------------------------------------------------    322
Gralnc.30550         ------------------------------------------------------------    313

PB.6980.1_gDNA       TTTATTTTACAAAAAGGAA-TGATCTTTGAGTAAAAAAAAAATTCCCAACTTTCTGTTTT    337
PB.6980.1            ------------------------------------------------------------    202
PB.23765.1           ------------------------------------------------------------    198
Ghrlnc.30744_At      ------------------------------------------------------------    457
Garlnc.26799         ------------------------------------------------------------    264
Gbalnc.26265_At      ------------------------------------------------------------    282
Gbalnc.64928_Dt      ------------------------------------------------------------    372
Ghrlnc.74560_Dt      ------------------------------------------------------------    322
Gralnc.30550         ------------------------------------------------------------    313

PB.6980.1_gDNA       TTGGATGAAATTTTTTGGCTTCACAAGCATCACCTGCTAAAACTATAAATTGGGTACTGA    397
PB.6980.1            ------------------------------------------------------------    202
PB.23765.1           ------------------------------------------------------------    198
Ghrlnc.30744_At      ------------------------------------------------------------    457
Garlnc.26799         ------------------------------------------------------------    264
Gbalnc.26265_At      ------------------------------------------------------------    282
Gbalnc.64928_Dt      ------------------------------------------------------------    372
Ghrlnc.74560_Dt      ------------------------------------------------------------    322
Gralnc.30550         ------------------------------------------------------------    313

PB.6980.1_gDNA       GTAT-TTTGTAATAATTTTGTCATGT--GTTTATAAATTCTAATGTTTGGACATGTGATT    454
PB.6980.1            ------------------------------------------------------------    202
PB.23765.1           ------------------------------------------------------------    198
Ghrlnc.30744_At      ------------------------------------------------------------    457
Garlnc.26799         ------------------------------------------------------------    264
Gbalnc.26265_At      ------------------------------------------------------------    282
Gbalnc.64928_Dt      ------------------------------------------------------------    372
Ghrlnc.74560_Dt      ------------------------------------------------------------    322
Gralnc.30550         ------------------------------------------------------------    313

PB.6980.1_gDNA       TTGTTTGGTTTTTCAGTTTCTTGGGATTTGTTGGATGGTAATACCAAAAAGGGAAGAAAA    512
PB.6980.1            ----------------TTTCTTGGGATTTGTTGGATGGTAATACCAAAAAGGGAAGAAAA    246
PB.23765.1           ----------------TTTCTTGGGATTTGTTGGGTGGTAATACCAAAAAGGGGAGAAAA    242
```

```
Ghrlnc.30744_At         ----------------TTTCTTGGGATTTGTTGGATGGTAATACCAAAAAGGGAAGAAAA     501
Garlnc.26799            ----------------TTTCTTGGGATTTGTTGGATGGTAATACCAAAAAGGGAAGAAAA     308
Gbalnc.26265_At         ----------------TTTCTTGGGATTTGTTGGATGGTAATACCAAAAAGGGAAGAAAA     326
Gbalnc.64928_Dt         ----------------TTTCTTGGGATTTGTTGGGTGGTAATACCAAAAAGGGGAGAAAA     416
Ghrlnc.74560_Dt         ----------------TTTCTTGGGATTTGTTGGGTGGTAATACCAAAAAGGGGAGAAAA     366
Gralnc.30550            ----------------TTTCTTGGGATTTGTTGGGTGGTAATACCAAAAAGGGGAGAAAA     357
                                        ********************************************


PB.6980.1_gDNA          CAGAGATGATTAATATA--TCTTTTTGCAAATTGAGATGTGGATCATGAT--GCTTTTGT     568
PB.6980.1               CAGAGATGATATATATATATCTTTTTGCAAATTGAGATGTGGATCATGATGTGCTTTTGT     304
PB.23765.1              CGGAGATGATTCATTTA--TCTTTTTGCAAATTGAGATGTGGATCATGAT--GCTTTTGT     298
Ghrlnc.30744_At         CAGAGATGATTAATATA--TCTTTTTGCAAATTGAGATGTGGATCATGAT--GCTTTTGT     557
Garlnc.26799            CAGAGATGATTCATATA--TCTTTTTGCAAATTGAGATGTGGATCATGAT--GCTTTTGT     364
Gbalnc.26265_At         CAGAGATGATTAATATA--TCTTTTTGCAAATTGAGATGTGGATCATGAT--GCTTTTGT     382
Gbalnc.64928_Dt         CGGAGATGATTCATTTA--TCTTTTTGCAAATTGAGATGTGGATCATGAT--GCTTTTGT     472
Ghrlnc.74560_Dt         CGGAGATGATTCATTTA--TCTTTTTGCAAATTGAGATGTGGATCATGAT--GCTTTTGT     422
Gralnc.30550            CGGAGATGATTCATTTA--TCTTTTTGCAAATTGAGATGTGGATCATGAT--GCTTTTGT     413
                        * ********  ** **  *******************************  ********

PB.6980.1_gDNA          AAATATTTGATTACAACTTAGATTTTGGGCAAATATA--AAATATAATATGATAATTATG     626
PB.6980.1               AAATATTTGATTACAACTTAGATTTTGGGCAATATAAAAAATATAATAATGATAATTATG     364
PB.23765.1              AAATATTTGATTACAACTTAGATTCTGGGCAAATATC--AAATGTAATATGATAATTATG     356
Ghrlnc.30744_At         AAATATTTGATTACAACTTAGATTTTGGGCAAATATA--AAATATAATATGATAATTATG     615
Garlnc.26799            AAATATTTGATTACAACTTAGATTTTGGGCAAATATA--AAATATAATATGATAATTATG     422
Gbalnc.26265_At         AAATATTTGATTACAACTTAGATTTTGGGCAAATATA--AAATATAATATGATAATTATG     440
Gbalnc.64928_Dt         ATATATTTGATTACAACTTAGATTCTGGGCAAATATC--AAATGTAATATGATAATTATG     530
Ghrlnc.74560_Dt         AAATATTTGATTACAACTTAGATTCTGGGCAAATATC--AAATGTAATATGATAATTATG     480
Gralnc.30550            AAATATTTGATTACAACCTAGATTCTGGGCAAATATC--AAATGTAACATGATAATTATG     471
                        * *************** ****** *******       **    *  ************

PB.6980.1_gDNA          TATTGGTAGAGCAAAAATGGAAAGGCTGTTTGCCCTCAATATAGAGCAATATCATCTT--     684
PB.6980.1               TATTGGTAGAGCAAAAATGGAAAGGCTGTTTGCCCTCAATATAGAGCAATATCATCTCTT     424
PB.23765.1              TATTGTTAGAGCAAAAATGGAAAGGCTGTTTGCCCTCAGTATAGAGCAATATCATCTT--     414
Ghrlnc.30744_At         TATTGGTAGAGCAAAAATGGAAAGGCTGTTTGCCCTCAATATAGAGCAATATCATCTT--     673
Garlnc.26799            TATTGGTAGAGCAAAAATGGAAAGGCTGTTTGCCCTCAATATAGAGCAATATCATCTT--     480
Gbalnc.26265_At         TATTGGTAGAGCAAAAATGGAAAGGCTGTTTGCCCTCAATATAGAGCAATATCATCTT--     498
Gbalnc.64928_Dt         TATTGTTAGAGCAAAAATGGAAAGGCTGTTTGCCCTCAGTATAGAGCAATATCATCTT--     588
Ghrlnc.74560_Dt         TATTGTTAGAGCAAAAATGGAAAGGCTGTTTGCCCTCAGTATAGAGCAATATCATCTT--     538
Gralnc.30550            TATTGTTAGAGCAAAAATGGAAAGGCTGTTTGCCCTCAATATAGAGCAATATCATCTT--     529
                        ***** ******************************** ******************

PB.6980.1_gDNA          TCTATACTGTATTCTATCCATTTATGTACCCCATATTTGATTAAATCTTAGATTTTGAGG     744
PB.6980.1               CTATAACTGTATTCTATCCATTTATGTACCCCATATTTGATTAAATCTTAGATTTTGAGG     484
PB.23765.1              TGTATACTGTATTTTATCCATTTATGTACCCCATCTTTGATTAAATCTTAGATTTTGAGG     474
Ghrlnc.30744_At         TCTATACTGTATTCTATCCATTTATGTACCCCATATTTGATTAAATCTTAGATTTTGAGG     733
Garlnc.26799            TCTATACTGTATTCTATCCATTTATGTACCCCATATTTGATTAAATCTTAGATTTTGAGG     540
Gbalnc.26265_At         TCTATACTGTATTCTATCCATTTATGTACCCCATATTTGATTAAATCTTAGATTTTGAGG     558
Gbalnc.64928_Dt         TGTATACTGTATTTTATCCATTTATGTACCCCATCTTTGATTAAATCTTAGATTTTGAGG     648
Ghrlnc.74560_Dt         TGTATACTGTATTTTATCCATTTATGTACCCCATCTTTGATTAAATCTTAGATTTTGAGG     598
Gralnc.30550            TGTATACTGTATTTTATCCATTTATGTACCCCATCTTTGATTAAATCTTAGATGTTGAGG     589
                             ******** ******************** ****************** ******

PB.6980.1_gDNA          GTATCAAATATAATGAAATTTATGTATCCTTAAAACCAAAAGGGAAAAGCTTTGATCCC-     803
PB.6980.1               GTATCAAATATAATGAAATTTATGTATCCTTAAAACCAAAAGGGAAAAGCTTTGATCCC-     543
PB.23765.1              GTATCAAATATAATGAAATTTATGTATCCTT-----------------------------     505
Ghrlnc.30744_At         GTATCAAATATAATGAAATTTATGTATCCTTAAAACCAAAAGGGAAAAGCTTTGATCCCA     793
Garlnc.26799            GTATCAAATATAATGAAATTTATGTATCCTTAAAACCAAAAGGGAAAAGCTTTGATCCCA     600
Gbalnc.26265_At         GTATCAAATATAATGAAATTTATGTATCCTTAAAACCAAAAGGGAAAAGCTTTGATCCCA     618
Gbalnc.64928_Dt         GTATCAAATATAATGAAATTTATGTATCCTTAAAACTAAAAGGGAAAAGCTTTGATCCCA     708
Ghrlnc.74560_Dt         GTATCAAATATAATGAAATTTATGTATCCTTAAAACTAAAAGGGAAAAGCTTTGATCCCA     658
Gralnc.30550            GTATCAAATATAATGAAATTTATGTATCCTTAAAACTAAAAGGGAAAAGCTTTGATCCCA     649
                        *******************************

PB.6980.1_gDNA          ------------------------------------------------------------     803
PB.6980.1               ------------------------------------------------------------     543
PB.23765.1              ------------------------------------------------------------     505
Ghrlnc.30744_At         CTTTGGAGCGAAATGGATGATTGTATACTTGTTACAGACA--------------------     833
Garlnc.26799            CTTTGGAGCGAAATGGATGATTGTATACTTGTTACAGACACTCCGCCGGAGACTTCATTT     660
Gbalnc.26265_At         CTTTGGAGCGAAATGGATGATTGTATACTTGTTACAGACACTCCGCCGGAGACTTCATTT     678
Gbalnc.64928_Dt         CTTTGGAGCGAAATGGATGATTGTATACTTGTTACAG-----------------------     745
Ghrlnc.74560_Dt         CTTTGGAGCGAAATGGATGATTGTATACTTGTTACAGACACTCCGCCGGAGACTTCATTT     718
Gralnc.30550            CTTTGGAGCGAAATGGATGATTGTATACTTGTTACTGACACTCCGCCGGAGACTTCATTT     709


PB.6980.1_gDNA          ------------------------------------------------------------     803
```

```
PB.6980.1           ------------------------------------------------------------     543
PB.23765.1          ------------------------------------------------------------     505
Ghrlnc.30744_At     ------------------------------------------------------------     833
Garlnc.26799        GACTCCACCGGATCCATCACCTTCGCCGTCATCTCTTCTTTTGGCACAAGATTTGTTCCG     720
Gbalnc.26265_At     GACTCCAC----------------------------------------------------     686
Gbalnc.64928_Dt     ------------------------------------------------------------     745
Ghrlnc.74560_Dt     GACTCCACTG-ATCCATCACCTTCGCCGTCATCTCTTCTTTTGGCACAAGATTTGTTCCG     777
Gralnc.30550        GACTCCACTGGATCCATCACCTTCGCCGTCATCTCTTCTTTTGGCACAAGATTTGTTCCG     769

PB.6980.1_gDNA      ------------------------------------------------------------     803
PB.6980.1           ------------------------------------------------------------     543
PB.23765.1          ------------------------------------------------------------     505
Ghrlnc.30744_At     ------------------------------------------------------------     833
Garlnc.26799        TTTAGTAATACCTAAATCCATTACCTCTAACAACTCAACAACTCCTATAAGACATGGTCA     780
Gbalnc.26265_At     ------------------------------------------------------------     686
Gbalnc.64928_Dt     ------------------------------------------------------------     745
Ghrlnc.74560_Dt     TTTAGTAGTACCTAAATCCATTACCTCTAACAACTCAACAACTCCTATAAGACATGGTCA     837
Gralnc.30550        TTTAGTAGTA--------------------------------------------------     779
```

Supplemental Figure S4. Sequence alignment of the two homologous lincRNAs predicted to be endogenous target mimics of miR2118e in *G. hirsutum* and *G. barbadense*. The two lincRNAs are identical except the 5' and 3' ends and a retained intron in Gbalnc.31516. The sequence highlighted in yellow is the binding site of miR2118e.

```
Ghrlnc.36832      ATTCATTATTAGGTTTTTGAAGTTCTTAAGTTTCAACGACCACCAGAAAATTTACAACAC      60
Gbalnc.31516      -----------GGTTTTTGAAGTTCTTAAGTTTCAACGACCACCAGAAAATTTACAACAC      49
                             *************************************************

Ghrlnc.36832      GTTTGGTTGGCATGAATAGCCAAACTATTCCTGGATGATTCGGTGATAAGCAGCTATTCA      120
Gbalnc.31516      GTTTGGTTGGCATGAATAGCCAAACTATTCCTGGATGATTCGGTGATAAGCAGCTATTCA      109
                  ************************************************************

Ghrlnc.36832      TTAGTTTGGTTCACTGAATCCTCCATTCGACAGAATTTCATTCATCTCCCATTCCGCCTC      180
Gbalnc.31516      TTAGTTTGGTTCACTGAATCCTCCATTCGACAGAATTTCATTCATCTCCCATTCCGCCTC      169
                  ************************************************************

Ghrlnc.36832      TTCCCGTAATAGCCATTCCCCACCTGATTTAAAGAATAGCAATACAGAGCCTCCACCGAA      240
Gbalnc.31516      TTCCCGTAATAGCCATTCCCCACCTGATTTAAAGAATAGCAATACAGAGCCTCCACCGAA      229
                  ************************************************************

Ghrlnc.36832      TAAGAAAAAAAACATAACCTCCCATTCTTGCCCTTTACCACTCTCCCTCAGCAAACACCT      300
Gbalnc.31516      TAAGAAAAAAAACATAACCTCCCATTCTTGCCCTTTACCACTCTCCCTCAGCAAACACCT      289
                  ************************************************************

Ghrlnc.36832      CAAGCCACCACACCCCAACCCTGTCTCCTAGTTCTCGTTTAGTTATTCTCATCCCACGAC      360
Gbalnc.31516      CAAGCCACCACACCCCAACCCTGTCTCCTAGTTCTCGTTTAGTTATTCTCATCCCACGAC      349
                  ************************************************************

Ghrlnc.36832      CATCATCGACAGAGATCTCGACATTATAAAAAGCATCCCCAAAGTTGAGACGAGTAAG--      420
Gbalnc.31516      CATCATCGACAGAGATCTCGACATTATAAAAAGCATCCCCAAAGTTGAGACGAGTAAGGT      409
                  **********************************************************

Ghrlnc.36832      ------------------------------------------------------------      420
Gbalnc.31516      TATTATTTCCCTCAATCTCACCTCCATTGTGAGAATTTCATCTCTTTACTTCTCTGCTTG      469

Ghrlnc.36832      ------------------------------------------------------------      420
Gbalnc.31516      TATTTCACTAGAAAGGGGTTGTGAAAAAAATGAGTTTTCGAAAAAAAATTGGTCAACTGG      529

Ghrlnc.36832      ------------------------------------------------------------      420
Gbalnc.31516      TTGATCCGATTATTTTAGGGCAATTTAGGGTTAGAAAGCTAAGCTTGGTTTATTCGAATT      589

Ghrlnc.36832      ------------------------------------------------------------      420
Gbalnc.31516      GTTGGGCATTTCTGTTTATTGTAAGGGATTTCAGGTTGCTTGTCTCAATAGTAAGAGATT      649

Ghrlnc.36832      ----------GTTGGAATTTGTTGTTTGTGGTAAATGGGTTGAAGGGGTTTTGGATTTTG      468
Gbalnc.31516      TCAATTTCAGGTTGGAATTTGTTGTTTGTGGTAAATGGGTTGAAGGGGTTTTGGATTTTG      709
                            **************************************************

Ghrlnc.36832      AGCTTTGAATCAGTTATAGAGTTTTTGAGCAAGAAAAAGAAAAAGAAAATAGAGGCGGGG      528
Gbalnc.31516      AGCTTTGAATCAGTTATAGAGTTTTTGAGCAAGAAAAAGAAAAAGAAAATAGAGGCGGGG      769
                  ************************************************************

Ghrlnc.36832      GAGATGAGGAAATCGTTTTACTGGCTCACATGCTCAAACTTATGATACTTTGAAGGGCAA      588
Gbalnc.31516      GAGATGAGGAAATCGTTTTACTGGCTCACATGCTCAAACTTATGATACTTTGAAGGGCAA      829
                  ************************************************************

Ghrlnc.36832      CAGTGGTGGTCAATCATGTATGCTCAGCCTATCAGAAGATAATATCCTGGACACTGTGGG      648
Gbalnc.31516      CAGTGGTGGTCAATCATGTATGCTCAGCCTATCAGAAGATAATATCCTGGACACTGTGGG      889
                  ************************************************************

Ghrlnc.36832      AGTCATTTGTGTTGACACTGAAAGAAATATAGCATAGGAGCCTCCAGTGGTGGTATTGTA      708
Gbalnc.31516      AGTCATTTGTGTTGACACTGAAAGAAATATAGCATAGGAGCCTCCAGTGGTGGTATTGTA      949
                  ************************************************************

Ghrlnc.36832      CTGAAGGTCTCTGGTCGTGTGGGATTAGCAGCAATGTATGGTGCAGGCTGTTGGGCCTCC      768
Gbalnc.31516      CTGAAGGTCTCTGGTCGTGTGGGATTAGCAGCAATGTATGGTGCAGGCTGTTGGGCCTCC      1009
                  ************************************************************

Ghrlnc.36832      TCAAAAGGACCCTTTGGGGCTCGTTTCATAGTTGGTTGTTGTGTTAGCGGTGCTGGAGAA      828
Gbalnc.31516      TCAAAAGGACCCTTTGGGGCTCGTTTCATAGTTGGTTGTTGTGTTAGCGGTGCTGGAGAA      1069
```

```
                  ************************************************************

Ghrlnc.36832      CACCTAATGAAAGGATTTTCAGCTCGGGAGTGCTGTGTCTCATCGTCACTGTAGGATTCT        888
Gbalnc.31516      CACCTAATGAAAGGATTTTCAGCTCGGGAGTGCTGTGTCTCATCGTCACTGTAGGATTCT        1129
                  ************************************************************

Ghrlnc.36832      TTTGGATTTATATGACTAACAGGAATTTGTTCCCTTTACATGAAGAAAGTTTTAGTTATC        948
Gbalnc.31516      TTTGGATTTATATGACTAACAGGAATTTGTTCCCTTTACATGAAGAAAGTTTTAGTTATC        1189
                  ************************************************************

Ghrlnc.36832      CTAAATGGACCTTGTCGAGTTAGGTTGTCTCTGCTTGTATCTCTTTTTATTAATACAAAA        1008
Gbalnc.31516      CTAAATGGACCTTGTCGAGTTAGGTTGTCTCTGCTTGTATCTCTTTTTATTAATACAAAA        1249
                  ************************************************************

Ghrlnc.36832      GTAGTATAATAGGATACGATCTTTTGAAACATTAATTGTCTACTTGATTCTGAGCATGGG        1068
Gbalnc.31516      GTAGTATAATAGGATACGATCTTTTGAAACATTAATTGTCTACTTGATTCTGAGCATGGG        1309
                  ************************************************************

Ghrlnc.36832      TGTTGAAATCGGTAAGGGTCTATGTTTGGTGAGAACTAGAAGTGATAAATCATATAATAG        1128
Gbalnc.31516      TGTTGAAATCGGTAAGGGTCTATGTTTGGTGAGAACTAGAAGTGATAAATCATATAATAG        1369
                  ************************************************************

Ghrlnc.36832      AAGTTGAATTTGTTCGATCTTAAATTATTTGCTTAAATTCGAAGATTTATCATCTTTGAT        1188
Gbalnc.31516      AAGTTGAATTTGTTCGATCTTAAATTATTTGCTTAAATTCGAAGATTTATCATCTTTGAT        1429
                  ************************************************************

Ghrlnc.36832      CCCATTTAGAAT        1200
Gbalnc.31516      CC----------        1431
                  **
```

Supplemental Figure S5. Sequence alignment of the two homologous endogenous target mimicries of miR2118e and their homologous PacBio full-length cDNAs. The sequence shown in red is the miR2118e binding site.

```
PB.25815.1       ------------------------------------------------------------        0
PB.13303.1       ------------------------------------------------------------        0
PB.8185.1        ------------------------------------------------------------        0
Ghrlnc.36832     ATTCATTATTAGGTTTTTGAAGTTCTTAAGTTTCAACGACCACCAGAAAATTTACAACAC        60
Gbalnc.31516     -----------GGTTTTTGAAGTTCTTAAGTTTCAACGACCACCAGAAAATTTACAACAC        49

PB.25815.1       ------------------------------------------------------------        0
PB.13303.1       ---------------------------ATTCCGGATGATTAGGTGATAAGTAGCTATTCA        33
PB.8185.1        -----------------------------------------GGTGATAAGCAGCTATTCA        19
Ghrlnc.36832     GTTTGGTTGGCATGAATAGCCAAACTATTCCTGGATGATTCGGTGATAAGCAGCTATTCA        120
Gbalnc.31516     GTTTGGTTGGCATGAATAGCCAAACTATTCCTGGATGATTCGGTGATAAGCAGCTATTCA        109

PB.25815.1       -----------------ATCTTCCATTCGACAGAATTTCATTCATCTTCC-ATTCCGCCT        42
PB.13303.1       TTAGTTTGGTTCACCGAATCCTCCATTCGACAGGATTTCATTCATCTCCC-ATTCCGTCT        92
PB.8185.1        TTAGTTTAATTCACCGAATCTCCA-TTCGACAGAATTTTATTCATCTCCCATTCCGCCCT        78
Ghrlnc.36832     TTAGTTTGGTTCACTGAATCCTCCATTCGACAGAATTTCATTCATCTCCC-ATTCCGCCT        179
Gbalnc.31516     TTAGTTTGGTTCACTGAATCCTCCATTCGACAGAATTTCATTCATCTCCC-ATTCCGCCT        168
                                  ***  *  ******** **** ******** **  * *   **

PB.25815.1       CCTCCTGTAATAGCCATTCCCTACCTGATCTAAAGAATAGCAATACAGAGCCTCCACCGA        102
PB.13303.1       TCTCCCATAATAGCCATTCCCCACCTGATCTAAAGAATAGCAATACTGAGCCTCCATCGG        152
PB.8185.1        CTACCCATAATAGCCATTCCCCACCTGATCTAAAGAATAGCAATACAGAGCCTCTACCGA        138
Ghrlnc.36832     CTTCCCGTAATAGCCATTCCCCACCTGATTTAAAGAATAGCAATACAGAGCCTCCACCGA        239
Gbalnc.31516     CTTCCCGTAATAGCCATTCCCCACCTGATTTAAAGAATAGCAATACAGAGCCTCCACCGA        228
                    **  ************** ******* **************** ******* * **

PB.25815.1       ATAAGAAAAAAAAACATAACCTCCCATTCTTGCGCCTTTACCACTCTTCCTCAGCAAACA        162
PB.13303.1       ATAAGAAAAAAAAACATAA-CCT-CCCATTCTTGCCCTTTACCACTCTTCCTCAGCAAACA        210
PB.8185.1        ATAAGAAAA-AAACATAA-CCT-CCCATTCTTGCCCTTTACCATTCTCCCTCAGCAAAC-        194
Ghrlnc.36832     ATAAGAAAAAAAAACATAA-CCT-CCCATTCTTGCCCTTTACCACTCTCCCTCAGCAAACA        297
Gbalnc.31516     ATAAGAAAAAAAAACATAA-CCT-CCCATTCTTGCCCTTTACCACTCTCCCTCAGCAAACA        286
                 ********* ***    * *** **  *  *   ********* *** ***********

PB.25815.1       CCTCAAGCCACCACACCCCAACCCTATCTCCTAGTTCTCGTTTAGTTATTCTCATCCCAC        222
PB.13303.1       CCTCAAGCCACCACACACCAGCCGTG----------------------------------        236
PB.8185.1        ACTCAAGCCACCACACCTCAACCTTGTCTCATAGTTCTCGTTTAGTTATTGTTATCCCAG        254
Ghrlnc.36832     CCTCAAGCCACCACACCCCAACCCTGTCTCCTAGTTCTCGTTTAGTTATTCTCATCCCAC        357
Gbalnc.31516     CCTCAAGCCACCACACCCCAACCCTGTCTCCTAGTTCTCGTTTAGTTATTCTCATCCCAC        346
                  ***************  ** ** *

PB.25815.1       AACCATCTTCGACAAAGATCTCGACATTGTAAAAAGCG----------------------        260
PB.13303.1       ------------------------------------------------------------        236
PB.8185.1        GACCATTATAGACAAAGATCTCGACATTGTAAAAAGCTTCCCCAAAGTTGAGACGAGCTC        314
Ghrlnc.36832     GACCATCATCGACAGAGATCTCGACATTATAAAAAGCATCCCCAAAGTTGAGACGAGTAA        417
Gbalnc.31516     GACCATCATCGACAGAGATCTCGACATTATAAAAAGCATCCCCAAAGTTGAGACGAGTAA        406

PB.25815.1       ------------------------------------------------------------        260
PB.13303.1       ------------------------------------------------------------        236
PB.8185.1        GACCTTTGCTTACTGAAGCAATTGAACATCA---AGACTTTGATATGTTGGTGGATCCGA        371
Ghrlnc.36832     G-----------------------------------------------------------        420
Gbalnc.31516     GGTTATTATTTCCCTCAATCTCACCTCCATTGTGAGAATTTCATCTCTTTACTTCTCTGC        466

PB.25815.1       ------------------------------------------------------------        260
PB.13303.1       ------------------------------------------------------------        236
PB.8185.1        GGCTGGAAAAGAACTACGTGGATCACGAAAT------GTTTCGA--------------AT        411
Ghrlnc.36832     ------------------------------------------------------------        420
Gbalnc.31516     TTGTATTTCACTAGAAAGGGGTTGTGAAAAAAATGAGTTTTCGAAAAAAAATTGGTCAAC        526

PB.25815.1       ------------------------------------------------------------        260
PB.13303.1       ------------------------------------------------------------        236
PB.8185.1        TTGATG------TTAATAATGGTAAACAAAGGGCTACAAGATTTAGTTATGTTGCTTACG        465
Ghrlnc.36832     ------------------------------------------------------------        420
Gbalnc.31516     TGGTTGATCCGATTATTTTAGGGCAATTTAGGGTTAGAAAGCTAAGCTTGGTTTATTCGA        586
```

```
PB.25815.1        ------------------------------------------------------------      260
PB.13303.1        ------------------------------------------------------------      236
PB.8185.1         T--------TCTCATTATGTATGCCAAAATATTGCAGACTGGATGGCAATCTGTAATGAG      517
Ghrlnc.36832      ------------------------------------------------------------      420
Gbalnc.31516      ATTGTTGGGCATTTCTGTTTATTGTAAGGGATTTCAGGTTGCTTGTCTCAAT----AGTA      642

PB.25815.1        ------------------------------------------------------------      260
PB.13303.1        ---------------------------------------------------------TCT      239
PB.8185.1         AATGAGTGCAAACTTACAGGACCTATTTCTATATGAGGCATTTTTGTATTATAATCATCT      577
Ghrlnc.36832      -------------------GTTGGAATTTGTTGTTTGTGGTAAATGGGTTGAAGGGGTTT      459
Gbalnc.31516      AGAGATT--TCAATTTCAGGTTGGAATTTGTTGTTTGTGGTAAATGGGTTGAAGGGGTTT      700

PB.25815.1        ------------------------------------------------------------      260
PB.13303.1        C------CTAGTTTTCGTTTAGTTATTCTCATC-CCACGACCATCATCGACAGAGATCTC      292
PB.8185.1         -------TCTTCTGGTGGTTAGTTAAAATTTTATGATATATAAAAAATTTATGTTATACT      630
Ghrlnc.36832      TGGATTTTGAGCTTTGAATCAGTTATAGAGTTTTTGAGCAAGAAAAAGAAAAAGAAAATA      519
Gbalnc.31516      TGGATTTTGAGCTTTGAATCAGTTATAGAGTTTTTGAGCAAGAAAAAGAAAAAGAAAATA      760

PB.25815.1        ----------------------TCCCTAAAGTTGAACGAGCTCACATGCTCAAACTTGTG      298
PB.13303.1        GACATTATAAAGCTTCCCTAAAGTTGAGAC----GAG---CTCACATTCTCAAACTTGTG      345
PB.8185.1         AATTTTTTCAATATGAGTGCAATTTTATGTTGTGTGTTTAAGGACTTGTAAGAGATTATT      690
Ghrlnc.36832      GAGGCGGGGGAGATGAGGAAATCGTTTTAC----TGG---CTCACATGCTCAAACTTATG      572
Gbalnc.31516      GAGGCGGGGGAGATGAGGAAATCGTTTTAC----TGG---CTCACATGCTCAAACTTATG      813
                                                             ** *     *  ** *

PB.25815.1        ATACTTTGGAGGACAACAGTGGTGGTCAATCATGCGTGGTCAATCATTTGATGCCCAGCC      358
PB.13303.1        ATACTTTGGAGGACAACAGTGGTGGTCAATCA---------------TGTATGCTCAGCC      390
PB.8185.1         CTCCTTGTTTTGATATC--------ACAGACT---------------ACAATGGTATGGC      727
Ghrlnc.36832      ATACTTTGAAGGGCAACAGTGGTGGTCAATCA---------------TGTATGCTCAGCC      617
Gbalnc.31516      ATACTTTGAAGGGCAACAGTGGTGGTCAATCA---------------TGTATGCTCAGCC      858
                   * ***     *  * *         **  *                   ***    * *

PB.25815.1        CATCACAAGACAATATCATGGACACTGTGGGAGTCATTTGGGTTGACACTGAAGGAAATA      418
PB.13303.1        CATCAGAAGACAATATCATGGACACTGTGGGAGTCATTTGTTTTTAACACTGAAGGAAAT      450
PB.8185.1         TTTGGGGAATCAATTTATGGGTCTTTTCTTAGTTCATTCCTC---------TTTGAGGCT      778
Ghrlnc.36832      TATCAGAAGATAATATCCTGGACACTGTGGGAGTCATTTGTGTTGACACTGAAAGAA-AT      676
Gbalnc.31516      TATCAGAAGATAATATCCTGGACACTGTGGGAGTCATTTGTGTTGACACTGAAAGAA-AT      917
                    *    *   *** *   ** *  *       *****                *

PB.25815.1        TAGCATCAGGAGCCTCCAGGCAGCGGTATTGCACTGAAGGTCTCTGTTCGTATGGGATTA      478
PB.13303.1        ATAGCATC-----------AAGAGCCTCCAGCGGTGAAGGTCTCCAGTCGTGTGAGATTA      499
PB.8185.1         ATAATTTA--TGAGACACTTTTTCCCACAATTGTGACAGGTCTCTGGTCATGTGGGATTA      836
Ghrlnc.36832      ATAGCATA-GGAGCCTCCAGTGGTGGTATTGTACTGAAGGTCTCTGGTCGTGTGGGATTA      735
Gbalnc.31516      ATAGCATA-GGAGCCTCCAGTGGTGGTATTGTACTGAAGGTCTCTGGTCGTGTGGGATTA      976
                                                       *******   ** * ** *****

PB.25815.1        GCAGCAATGTATGGTGCAGGCTGTTGGGGCCTCCTCAAAAGGACCCTTTGGGGCTTCTTT      538
PB.13303.1        GCAACAATGTACGGTGCAGGCTGTTGGG-CCTCCTCAAAAGGACCTTTTGGGGCTCCTTT      558
PB.8185.1         GCAGCAATGTATGGTGCAGGCTGTTGGG-CATCCTCAAAAGGACCTTTTGGGGCTCCTTT      895
Ghrlnc.36832      GCAGCAATGTATGGTGCAGGCTGTTGGG-CCTCCTCAAAAGGACCCTTTGGGGCTCGTTT      794
Gbalnc.31516      GCAGCAATGTATGGTGCAGGCTGTTGGG-CCTCCTCAAAAGGACCCTTTGGGGCTCGTTT      1035
                  *** ******* **************** * ************** *********  ***

PB.25815.1        CATAGTTGGTTGTTGTGTTAGCGGTGCTGGGAGAATACCTAATGAAAAGATTTGCAGCTC      598
PB.13303.1        CATAGTTGCTTGTTGTATTAGCAGTGCTGGAGAAA-CACTAATGAAAGGATTTGCAGCTC      617
PB.8185.1         CACAGTTGGTTGTTGTGTTAGCGATGCTGGAGAAC-ACCTAATGAAAAGATTTGCAGCTC      954
Ghrlnc.36832      CATAGTTGGTTGTTGTGTTAGCGGTGCTGGAGAAC-ACCTAATGAAAGGATTTTCAGCTC      853
Gbalnc.31516      CATAGTTGGTTGTTGTGTTAGCGGTGCTGGAGAAC-ACCTAATGAAAGGATTTTCAGCTC      1094
                  ** ***** ******* *****  ******   *    ********* ***** ******

PB.25815.1        GGGAGTGCGTGTGTCTCATCGTCACTATAAGATTCTTTTGGATTTATATGCCTAATAGGA      658
PB.13303.1        GGGA-GTGCTGTGTCTCATCGTCACTGTAAGATTCTTTTGGATTTATATGCCTAACAGGA      676
PB.8185.1         GGGA-GTGCTGTGTCTCATCGTACT-GTAAGATTCTTTTGGATTTATATGCCTAACATGA      1012
Ghrlnc.36832      GGGA-GTGCTGTGTCTCATCGTCACTGTAGGATTCTTTTGGATTTATATGACTAACAGGA      912
Gbalnc.31516      GGGA-GTGCTGTGTCTCATCGTCACTGTAGGATTCTTTTGGATTTATATGACTAACAGGA      1153
                  ****     *************     ** ******************** **** * **

PB.25815.1        ATTTGCTCCCTTTACATGAAGAAAGTTTTAGTTATCCTAAATGGACCTTGTTGAGTTAGG      718
PB.13303.1        ATTTGTTCCCTTTACATGAAGAAAGTTTCAGTTATCCTAAATGGACCTTGTTGAGTTAGG      736
PB.8185.1         ATTTGATCCTT--TACATGAGAAAGTTTCAGTTATCCTAAATGGACCTTGTTGAGTTAGG      1070
Ghrlnc.36832      ATTTGTTCCCTTTACATGAAGAAAGTTTTAGTTATCCTAAATGGACCTTGTCGAGTTAGG      972
Gbalnc.31516      ATTTGTTCCCTTTACATGAAGAAAGTTTTAGTTATCCTAAATGGACCTTGTCGAGTTAGG      1213
                  ***** *** *        ********* ********************** ********
```

```
PB.25815.1      TTGTCTCTGCTTGTATCGCTTTCTATTAATACAAAAGTAGTATAATAGGATGGCGATCTT   778
PB.13303.1      TTGTCTCTGCTTTTATCTCTTTCTATTAATACAAAAGTAGTATAGTAGGATAC-GATCTT   795
PB.8185.1       TTGTCTCTGCTTGTATCTCTTTCTATTAATACAAAAGTAGTATAATAGGATAC-GATCTT   1129
Ghrlnc.36832    TTGTCTCTGCTTGTATCTCTTTTTATTAATACAAAAGTAGTATAATAGGATAC-GATCTT   1031
Gbalnc.31516    TTGTCTCTGCTTGTATCTCTTTTTATTAATACAAAAGTAGTATAATAGGATAC-GATCTT   1272
                ************ **** **** ********************* ******   ******

PB.25815.1      TTGAAACATTAATTATCTACTTGATTCCGAGCATGGGTGTGTTGAAATCGGTAACGGTCT   838
PB.13303.1      TTAAAACATTAATTGTCTACTTGATTCCGAGCATGGGTG--TTGAAATCGGTAACGGTCT   853
PB.8185.1       TTAAAACATTAATTGTCTACTTGATTCAGAGCATGGGTG--TTGAAATTGGTAACGGTCT   1187
Ghrlnc.36832    TTGAAACATTAATTGTCTACTTGATTCTGAGCATGGGTG--TTGAAATCGGTAAGGGTCT   1089
Gbalnc.31516    TTGAAACATTAATTGTCTACTTGATTCTGAGCATGGGTG--TTGAAATCGGTAAGGGTCT   1330
                ** *********** ************ ***********  ******* ***** *****

PB.25815.1      ATGCTTGGTGAGAACTTAGAAGTGATAAATCATATAATAGAAGTTGAATTTGTTCAATCT   898
PB.13303.1      ATGTTTGGTGAGAACTAGAAGTGATAAATCATATATATAGAAGTTGAATTTGTTCGATCT   913
PB.8185.1       ATGCTTGGTGAGACTA-GAAGTGATAAATCATAT-AATAGAAGTTGAATTTGTTCGATCT   1245
Ghrlnc.36832    ATGTTTGGTGAGAACTAGAAGTGATAAATCATATAA-TAGAAGTTGAATTTGTTCGATCT   1148
Gbalnc.31516    ATGTTTGGTGAGAACTAGAAGTGATAAATCATATAA-TAGAAGTTGAATTTGTTCGATCT   1389
                *** *********      *      **         ****************** ****

PB.25815.1      TAAATTATTTGCTTAAATTTGAAGCTATAGAAATTGCAGCTGCGTACTCATCCTTGTCTT   958
PB.13303.1      TAAATTATTTGCTTAAATTCGAAGATTTATCATCTTTGATCCCATTTAGAATATTTAAGG   973
PB.8185.1       TAAATTATTTGCTTAAATTCGAAGATTTATCATCTTTGATCCCATTTAGAATATTTAAGG   1305
Ghrlnc.36832    TAAATTATTTGCTTAAATTCGAAGATTTATCATCTTTGATCCCATTTAGAAT--------   1200
Gbalnc.31516    TAAATTATTTGCTTAAATTCGAAGATTTATCATCTTTGATCC------------------   1431
                ******************* **** * **  *  *

PB.25815.1      TTGGGATAGGATATTT----------------------------------TGGAAGTGGC   984
PB.13303.1      TAAGCTAAAAAGAGAAATGAATGACACAAGTAAATAATTTTG-GCTCCTTTGAAACATAC   1032
PB.8185.1       TAAGCTAAAAGAGAAATG-AAGGACACAAGTAAATAATTTTGGCTACCTTTGAAAAATAC   1364
Ghrlnc.36832    ------------------------------------------------------------   1200
Gbalnc.31516    ------------------------------------------------------------   1431

PB.25815.1      ATAGGAA---------------------------GGCCTAAG------------------   999
PB.13303.1      ATTAGATTCATTGCAAT-------------------------------TTGCATGAAAGT   1061
PB.8185.1       ATTAGATTCATTGCAATTTGCAATACTTGGGGTACTAATAAGGTCATTTTCATGCTTAGT   1424
Ghrlnc.36832    ------------------------------------------------------------   1200
Gbalnc.31516    ------------------------------------------------------------   1431

PB.25815.1      -----GTATGATGCTTGCAATTAAAACATGCATGTTTTTGGAGTAGATGGTATATTCATT   1054
PB.13303.1      TTACAGGCAGGTCCTGCCTCTGCTTGCATGAAAGTTCTTTGCTCCGTTGCTCAAGATAGT   1121
PB.8185.1       TTATAGGCAGGTCCTGCCTCTGCTTGCATGAAAGTTCTTCGCTCTATTGCTCAAGATAGT   1484
Ghrlnc.36832    ------------------------------------------------------------   1200
Gbalnc.31516    ------------------------------------------------------------   1431

PB.25815.1      GATGTAGCAAA---TTATAAGTGTG---TGGTTAAGAT----------------------   1086
PB.13303.1      AATCAAGCTGATACTGATAAAAGTGATGGGATTTTAATCGTGCAAGCGGGTGCTCCTATC   1181
PB.8185.1       AATCAAGCTGATACTGATAAAAGTGCTGGGATTTTAATCGTGCAAGCGGATGCTCCTATT   1544
Ghrlnc.36832    ------------------------------------------------------------   1200
Gbalnc.31516    ------------------------------------------------------------   1431

PB.25815.1      -----------------------------------------ACTGCATTAATTATGTATG   1105
PB.13303.1      AGGGCGAGTTCTCAATTAAACAACAATATATCTTTAAAAAAAATACATTTATCGGATTAG   1241
PB.8185.1       AGGGCGAGATCTCAATTAAACCACAATACATCTTTCG-----------------------   1581
Ghrlnc.36832    ------------------------------------------------------------   1200
Gbalnc.31516    ------------------------------------------------------------   1431

PB.25815.1      GTTATGAAGT---------CCCAATTATTTCCAGCAATGCTTTGACAGCCATTAGAGATG   1156
PB.13303.1      TTTTTATATGTGAGTTTATTGGTTTAGGTTCCAGGAAATCCTCCAAAACTGAAAGCTATA   1301
PB.8185.1       ------------------------------------------------------------   1581
Ghrlnc.36832    ------------------------------------------------------------   1200
Gbalnc.31516    ------------------------------------------------------------   1431

PB.25815.1      AAAATATTT---ATGTA-----CTC------------TCTATCTGTTGTTTATGCTTTTT   1196
PB.13303.1      TAGAAATTGCAGCTGCGTACTCATCCTTGTCTTTTGGGATAGGATATTTTGGAAGTGGCA   1361
PB.8185.1       ------------------------------------------------------------   1581
Ghrlnc.36832    ------------------------------------------------------------   1200
Gbalnc.31516    ------------------------------------------------------------   1431
```

```
PB.25815.1        TTTTCTTGATTCAGGTTTCTATTCTTAGGAGAAGTAAACAACAGAATAGAAGTGGAATCG    1256
PB.13303.1        TGGAACGGCCTAAGGTTTCTATTCTTAGAAAAAGTAAACAGCAGAATAGAAGTGGAATCG    1421
PB.8185.1         ------------------------------------------------------------    1581
Ghrlnc.36832      ------------------------------------------------------------    1200
Gbalnc.31516      ------------------------------------------------------------    1431

PB.25815.1        ATCATTTTGAAGCTCGAGTTGATGTTTCTACATGACTGGTTTTTGTTAACATGTTTTTTT    1316
PB.13303.1        ATCATTTTGAAGCTCAAGTTGATGTTTCTACATGACTGATTTTTGTTTAACCTATTTT--    1479
PB.8185.1         ------------------------------------------------------------    1581
Ghrlnc.36832      ------------------------------------------------------------    1200
Gbalnc.31516      ------------------------------------------------------------    1431

PB.25815.1        TTTAAGGATAAATTATCATATCAAATCAAATTCTCTCCAATTTACTTGGTTTCTTTGGGA    1376
PB.13303.1        TTCAAGGATAAATTACCATATCAA-ATCAAAACTCTCCAATTTACTTAGTTTCTTTGGGA    1538
PB.8185.1         ------------------------------------------------------------    1581
Ghrlnc.36832      ------------------------------------------------------------    1200
Gbalnc.31516      ------------------------------------------------------------    1431

PB.25815.1        GTGTATGTAGTACTATAAAGAGAAAGTAACTTGTCCCCCTTCGAGATTGCCATTTATTGT    1436
PB.13303.1        GTGTATGTAGTACTATAAAAGAAAGTA-ACCTGTCCCCCTTTGAGATTGTCATTTA-ATG    1596
PB.8185.1         ------------------------------------------------------------    1581
Ghrlnc.36832      ------------------------------------------------------------    1200
Gbalnc.31516      ------------------------------------------------------------    1431

PB.25815.1        GTTAATATCGATGAATAAAAGTTAAAGTGATCAAATCGATATGATCTTAACTCTCATC      1494
PB.13303.1        GTTAATATCGATGAATAAAAGTTAAAGTGATCAAATCGATATGATCTTAACCTT----      1650
PB.8185.1         ----------------------------------------------------------      1581
Ghrlnc.36832      ----------------------------------------------------------      1200
Gbalnc.31516      ----------------------------------------------------------      1431
```
